# First real-time imaging of bronchoscopic lung volume reduction by electrical impedance tomography

**DOI:** 10.1186/s12931-024-02877-0

**Published:** 2024-07-04

**Authors:** Vinicius Torsani, Paulo Francisco Guerreiro Cardoso, João Batista Borges, Susimeire Gomes, Henrique Takachi Moriya, Andrea Fonseca da Cruz, Roberta Ribeiro de Santis Santiago, Cristopher Kengo Nagao, Mariana Fernandes Fitipaldi, Marcelo do Amaral Beraldo, Marcus Henrique Victor Junior, Mikuláš Mlček, Paulo Manuel Pego-Fernandes, Marcelo Britto Passos Amato

**Affiliations:** 1 Divisao de Pneumologia, Instituto do Coracao, Hospital das Clinicas HCFMUSP, Faculdade de Medicina, Universidade de Sao Paulo, Sao Paulo, Brasil; 2 Division of Thoracic Surgery, Thoracic Surgery Research Laboratory (LIM 61), Instituto do Coracao, Hospital das Clinicas HCFMUSP, Faculdade de Medicina, Universidade de Sao Paulo, Sao Paulo, Brasil; 3 Institute of Physiology, First Faculty of Medicine, Charles University, Albertov 5, Prague, 128 00 Czech Republic; 4 Biomedical Engineering Laboratory, Escola Politecnica da Universidade de Sao Paulo, Sao Paulo, Brasil; 5 Department of Anesthesia, Critical Care and Pain Medicine, Harvard Medical School, Massachusetts General Hospital, Boston, MA USA

**Keywords:** Absorption atelectasis, Electrical impedance tomography, Emphysema, Endobronchial valves, Lung volume reduction

## Abstract

**Background:**

Bronchoscopic lung volume reduction (BLVR) with one-way endobronchial valves (EBV) has better outcomes when the target lobe has poor collateral ventilation, resulting in complete lobe atelectasis. High-inspired oxygen fraction (F_I_O_2_) promotes atelectasis through faster gas absorption after airway occlusion, but its application during BLVR with EBV has been poorly understood. We aimed to investigate the real-time effects of F_I_O_2_ on regional lung volumes and regional ventilation/perfusion by electrical impedance tomography (EIT) during BLVR with EBV.

**Methods:**

Six piglets were submitted to left lower lobe occlusion by a balloon-catheter and EBV valves with F_I_O_2_ 0.5 and 1.0. Regional end-expiratory lung impedances (EELI) and regional ventilation/perfusion were monitored. Local pocket pressure measurements were obtained (balloon occlusion method). One animal underwent simultaneous acquisitions of computed tomography (CT) and EIT. Regions-of-interest (ROIs) were right and left hemithoraces.

**Results:**

Following balloon occlusion, a steep decrease in left ROI-EELI with F_I_O_2_ 1.0 occurred, 3-fold greater than with 0.5 (*p* < 0.001). Higher F_I_O_2_ also enhanced the final volume reduction (ROI-EELI) achieved by each valve (*p* < 0.01). CT analysis confirmed the denser atelectasis and greater volume reduction achieved by higher F_I_O_2_ (1.0) during balloon occlusion or during valve placement. CT and pocket pressure data agreed well with EIT findings, indicating greater strain redistribution with higher F_I_O_2_.

**Conclusions:**

EIT demonstrated in real-time a faster and more complete volume reduction in the occluded lung regions under high F_I_O_2_ (1.0), as compared to 0.5. Immediate changes in the ventilation and perfusion of ipsilateral non-target lung regions were also detected, providing better estimates of the full impact of each valve in place.

**Trial registration:**

Not applicable.

**Supplementary Information:**

The online version contains supplementary material available at 10.1186/s12931-024-02877-0.

## Background

Bronchoscopic lung volume reduction (BLVR) with one-way endobronchial valves (EBV) is a minimally invasive endoscopic procedure that improves clinical outcomes in selected emphysema patients with severe hyperinflation [1, 2]. Best outcomes are achieved when atelectasis of the target lobe occurs after BLVR with EBV [3] and in the absence of collateral ventilation (CV) between the target and adjacent lobe(s), which favors lobar atelectasis [4].

In human lungs, the quantity and resistance of CV channels varies widely, requiring individual assessment, particularly when radiographic evaluation of the interlobar fissures between the target and adjacent lobes shows an integrity below 95% [5–7]. In such instances, the bronchoscopic assessment of CV is necessary by means of a balloon catheter connected to a dedicated device that measures real-time CV [4]. Once the negative collateral ventilation (CV-) is ascertained, the EBV valves are placed in the segmental bronchi of the target lobe.

Despite being the best candidates for BLVR with EBV, CV- patients have a 15 to 27% probability of developing a pneumothorax in the non-treated emphysematous lobe(s) after valve placement [8–10]. Approximately 86% of the pneumothorax events occur within 3 days following BLVR [10–12]. Parenchymal interdependence is thought to be the main physiological mechanism underlying such pneumothorax events. The mechanism involves the targeted lobar deflation, which may cause overinflation of ipsilateral lobe(s), resulting in a tear of emphysematous ipsilateral lobe(s), resulting in pneumothorax. There is an increased local strain after stress redistribution, particularly within adjacent non-target lobes [13].

Oxygen is rapidly absorbed through the alveolar-capillary barrier playing an important role in atelectasis formation in patients under general anesthesia, especially when associated with airway occlusion [14–16]. These conditions have similarities with the intended effect of EBV. However, the real-time kinetics of all the regional acute effects of the inspired oxygen fraction (F_I_O_2_) on BLVR with EBV are poorly understood. This would require a bedside monitoring tool capable of providing real-time and continuous information on regional lung volumes, or more precisely in the strain map of the lung after BLVR with EBV placement.

Electrical impedance tomography (EIT) is a non-invasive, bedside lung imaging tool that provides real-time and continuous information on regional changes in end-expiratory lung volume [17] and strain, as well as regional changes in lung ventilation and perfusion [18–20]. Changes in lung function during bronchoscopic procedures have also been monitored with EIT [21, 22]. Furthermore, EIT can also accurately detect the early onset of pneumothorax [23, 24].

This experimental study focused on using EIT imaging to investigate the real-time effects of different F_I_O_2_ on regional lung volumes and regional ventilation/perfusion during BLVR with EBV in a CV- animal model. We hypothesized that a higher F_I_O_2_ during airway occlusion induces greater effects in lung volume reduction, and such changes could be early and precisely detected by EIT, as well as the distinct responses on the non-targeted ipsilateral lung regions. We also present two illustrative clinical cases of BLVR with EBV. The information provided by EIT monitoring was relevant and timely related to key mechanisms that materialized in the post-BLVR evolution and to the clinical outcomes in these patients.

## Methods

Additional details on the study design and methods are provided as Supplementary Information (See Supplementary Information 1, Additional File 1). This study was approved by the ethics committee for experimental studies of the Faculdade de Medicina da Universidade de Sao Paulo, Sao Paulo, Brazil (CEUA 200 − 12), and by the ethics committee for clinical studies (CAAe 43250215.0.1001.5327 and CAPPesq 0689 − 11).

### Animal experiments

Six healthy female Landrace piglets were anesthetized. In a crossover study design, they were submitted to two occlusion methods (*Balloon* and *Valves*) and two F_I_O_2_ (0.5 and 1.0). The occlusion method order was fixed with *Balloon* first (See Supplementary Figure S1, Additional File 2). The F_I_O_2_ order was randomized.

### Balloon occlusion method

Bronchial occlusion of the left lower lobe (LLL) was achieved by the placement of an indwelling inflatable Chartis™ balloon-catheter (Chartis™, Pulmonx Inc. USA) under bronchoscopic view. The following time points were analyzed: ***Pre*** [under *standard mechanical ventilation (MV) settings*; See Supplementary Figure S1, Additional File 2]; ***Broncho*** (1 min after positioning the bronchoscope in the LLL bronchus); ***T0*** (when complete LLL bronchial occlusion with the balloon was achieved); and each minute from ***T1*** to ***T15*** (1 to 15 min of occlusion).

### Valves occlusion method

The bronchoscopic placement of one-way endobronchial valves EBV Zephyr™ 4.0–5.5 mm (Pulmonx Inc. USA) was carried out under bronchoscopic view and endobronchial valves were deployed in the segmental bronchi of the LLL. The following time points were analyzed: ***Pre*** (under *standard MV settings*); ***Broncho*** (1 min after positioning the bronchoscope in the LLL bronchus); ***T0*** (when complete LLL bronchial occlusion with the valves was achieved); and each minute from ***T1*** to ***T15*** (1 to 15 min of occlusion); ***T30*** and ***T45*** (30 and 45 min of occlusion).

### EIT

*Regional Lung Volumes and Regional Ventilation* functional lung images were generated [18, 25, 26]. Measurements were regional tidal impedance variation (TIV) and regional end-expiratory lung impedance (EELI) [17, 19]. Data were relative to ***Pre*** values. *Perfusion Distributions* were acquired as previously described [20]. The images were sub-segmented into two regions-of-interest (ROIs): *Right* and *Left* hemithorax.

### Computed tomography scans

Helical computed tomography (CT) scans and EIT imaging were simultaneously acquired in one piglet. The time points ***Pre***, each minute from ***T0*** to ***T5***, and ***T15*** were analyzed as regards to the CT-derived gas content. Dynamic contrast-enhanced CT perfusion distributions, as described in detail elsewhere [27, 28], were also acquired at the time points ***Pre*** and ***T15***, having corresponding EIT perfusion distributions.

### Local pocket pressure

During the *Balloon Occlusion Method*, a pressure transducer was connected to a hollow catheter to provide continuous recordings of the pressure changes in the air pocket distal to the occluded bronchi. This methodology has been described in detail previously [29, 30].

### Patients observations

The observations in the first patient focused on the CV evaluation to define the target lobe where CV was absent. The observations of the second patient focused on the periods before, during, and after the EBV Zephyr™ valve placement. EIT images were sub-segmented into the same ROIs as the piglets.

### Statistical analysis

The assumptions of a normal distribution in each group and the homogeneity of the variances between groups were evaluated with the Shapiro-Wilk and Levene tests. A two-way analysis of variance (ANOVA) was used for a two-factor analysis (group and time), and Bonferroni adjustment for multiple tests was applied for *post-hoc* comparisons. Statistical significance was considered for *p* values less than 0.05. Values presented are mean and SEM unless otherwise stated.

## Results

Additional details on the results are provided as Supplementary Information (See Supplementary Information S1, Additional File 1).

### Experimental data

The six piglets completed the study protocol and were included in the analyses. All EIT data on regional TIV and EELI were available for analysis. The individualized optimum positive end-expiratory pressure (PEEP) was 16 ± 2 cmH_2_O. The MV parameters, physiological measurements, and respiratory system mechanics for both occlusion methods are shown (See Supplementary Tables S1 and S2, Additional Files 3 and 4).

### Balloon occlusion method

**Fig. 1:**
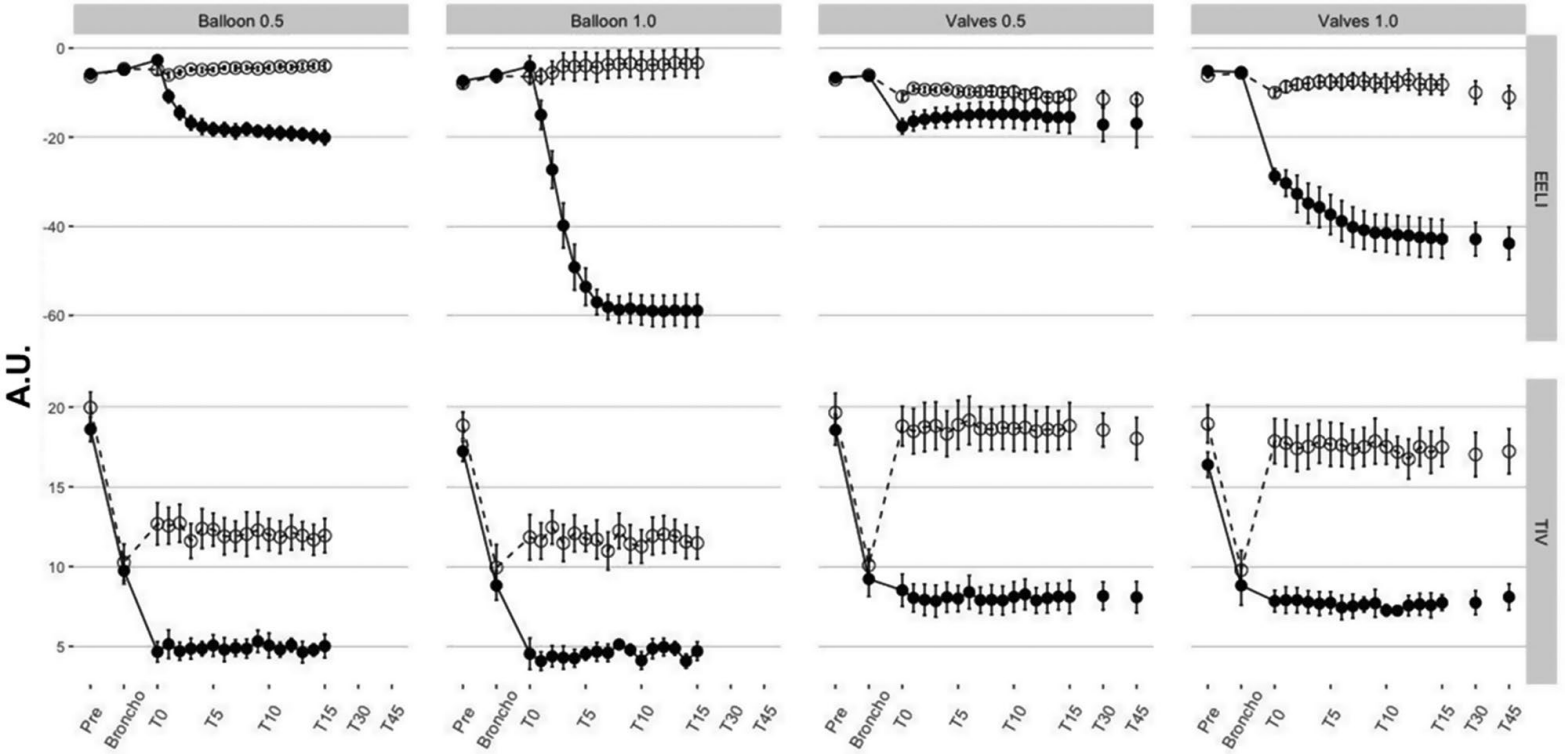
Regional lung volumes and regional ventilation by electrical impedance tomography. The changes in regional lung volumes and regional ventilation, by electrical impedance tomography (EIT), of both occlusion methods (*Balloon* and *Valves*) and inspired oxygen fractions (F_I_O_2_). Tidal impedance variation (TIV) is the regional impedance amplitude during a tidal breath (regional ventilation), and the regional end-expiratory lung impedance (EELI) is the regional impedance plethysmography baseline, a surrogate for regional end-expiratory lung volume. Time points of the *Balloon Occlusion* were ***Pre*** (under the *standard mechanical ventilation settings*); ***Broncho*** [1 min after positioning the bronchoscope in the left lower lobe (LLL) bronchus]; ***T0*** (complete LLL bronchus occlusion with the balloon); ***T1*** to ***T15*** (each minute from 1 to 15 min after occlusion). Time points of the *Valves Occlusion* were ***Pre*** (under the *standard mechanical ventilation settings*); ***Broncho*** (1 min after positioning the bronchoscope in the LLL bronchus); ***T0*** (completion of LLL bronchus occlusion); ***T1*** to ***T15*** (each minute from 1 to 15 min after occlusion); ***T30*** and ***T45*** (30 and 45-minute occlusion). EIT image was sub-segmented into two regions of interest (ROIs) corresponding to right and left hemithoraces. EIT data were relative to ***Pre*** values for each F_I_O_2_ and occlusion method combination. White circle: right hemithorax ROI; Black circle: left hemithorax ROI; A.U.: arbitrary units

The bronchoscope insertion did not affect regional EELI compared to the ***Pre*** values. Following balloon occlusion, there was a steep decrease of the EELI of the left ROI with F_I_O_2_ 1.0 (Fig. 1), which was 3-fold greater than in F_I_O_2_ 0.5 (*p* < 0.001). The EELI of the right ROI did not change over time in both F_I_O_2_. The TIV of both hemithoraces decreased to approximately half with the introduction of the bronchoscope. With the balloon occlusion, the left ROI presented an additional decrease and a contralateral increase during both F_I_O_2_ regimens (***Broncho*** vs. ***T0***: F_I_O_2_ 1.0, *p* < 0.01; F_I_O_2_ 0.5, *p* = 0.01). Then, the regional TIV of both hemithoraces remained unchanged for 15 min (Fig. 1).

### Valves occlusion method

Five animals received three valves; one required four valves to occlude the LLL segments. All valve deployments were in place within 3 min. The insertion of the bronchoscope in the LLL did not change regional EELI. Under the F_I_O_2_ 1.0, at ***T0*** and onwards, there was a progressive reduction of the left ROI EELI (***Pre*** vs. ***T0***: *p* < 0.01). The same did not occur with F_I_O_2_ 0.5. Regardless of the F_I_O_2_, the right ROI EELI showed no significant differences (Fig. 1). The TIV of both hemithoraces showed a 50% decrease after the insertion of the bronchoscope. During occlusion and onwards, this reduction was maintained in the TIV measurements of the left ROI. On the other hand, following the bronchoscope withdrawal, the right ROI’s TIV returned to ***Pre*** values and remained unchanged for 45 min (Fig. 1). The regional TIV data was not different between the two F_I_O_2_ regimens.

### CT and EIT images

**Fig. 2:**
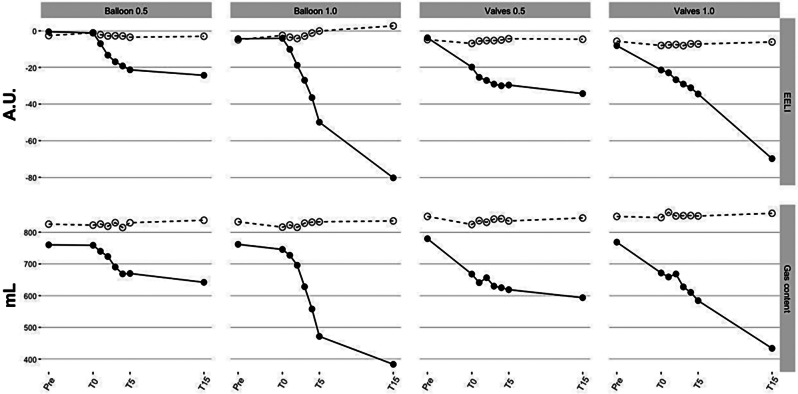
Regional end-expiratory lung impedance and computed tomography-derived gas content. Regional end-expiratory lung impedance (EELI) data by electrical impedance tomography (EIT) and computed tomography (CT)-derived gas content data from one animal of occlusion methods (*Balloon* and *Valves*), and fraction of inspired oxygen (F_I_O_2_) 0.5 and 1.0. EIT and CT images were sub-segmented into two regions-of-interest (ROIs): right and left hemithoraces. The EELI of the right ROI remained unchanged regardless of the F_I_O_2_. Conversely, the EELI of the left ROI containing the occluded left lower lobe showed a significant decrease under F_I_O_2_ 1.0 compared to 0.5. The CT-derived gas content data paralleled the EELI findings. White circle: right hemithorax ROI; Black circle: left hemithorax ROI; A.U: arbitrary units

**Fig. 3:**
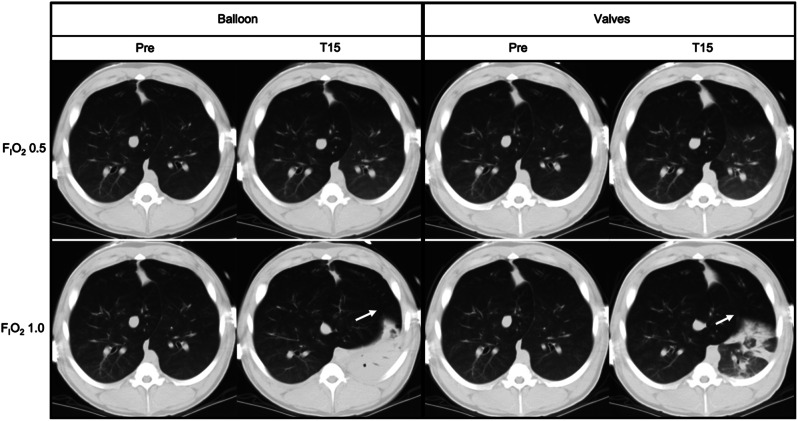
Computed tomography images. Computed tomography (CT) images from both occlusion methods and fraction of inspired oxygen (F_I_O_2_). Time points were ***Pre*** (under the *standard mechanical ventilation settings*) and ***T15*** (15-minute occlusion). The ***Pre*** vs. ***T15*** CT images were unequivocally different with F_I_O_2_ 1.0. In addition, the ***T15*** CT images with F_I_O_2_ 1.0 were also clearly different between the two occlusion methods. The F_I_O_2_ 1.0 resulted in complete atelectasis with the balloon and partial atelectasis with the valves. The images at ***T15*** with F_I_O_2_ 1.0 also show a visible expansion of the accessory lobe displaced towards the left hemithorax (white arrow), which was more prominent in the *Balloon Occlusion Method*

The CT and EIT data acquired in one animal illustrates and corroborates the data observed in the other animals. The EELI of the right ROI remained unchanged regardless of the F_I_O_2_. Conversely, the EELI of the left ROI, which contains the occluded LLL, decreased and showed a significantly greater decrease with F_I_O_2_ 1.0 when compared to F_I_O_2_ 0.5 (Fig. 2). The CT-derived gas content data paralleled the EELI findings consistently. The ***Pre*** vs. ***T15*** CT images showed the faster evolution of atelectasis under F_I_O_2_ 1.0 (Fig. 3). In addition, the ***T15*** CT images with F_I_O_2_ 1.0 were also clearly different between the two occlusion methods (Fig. 3). F_I_O_2_ 1.0 resulted in complete atelectasis with the balloon and partial atelectasis with the valves.

**Fig. 4:**
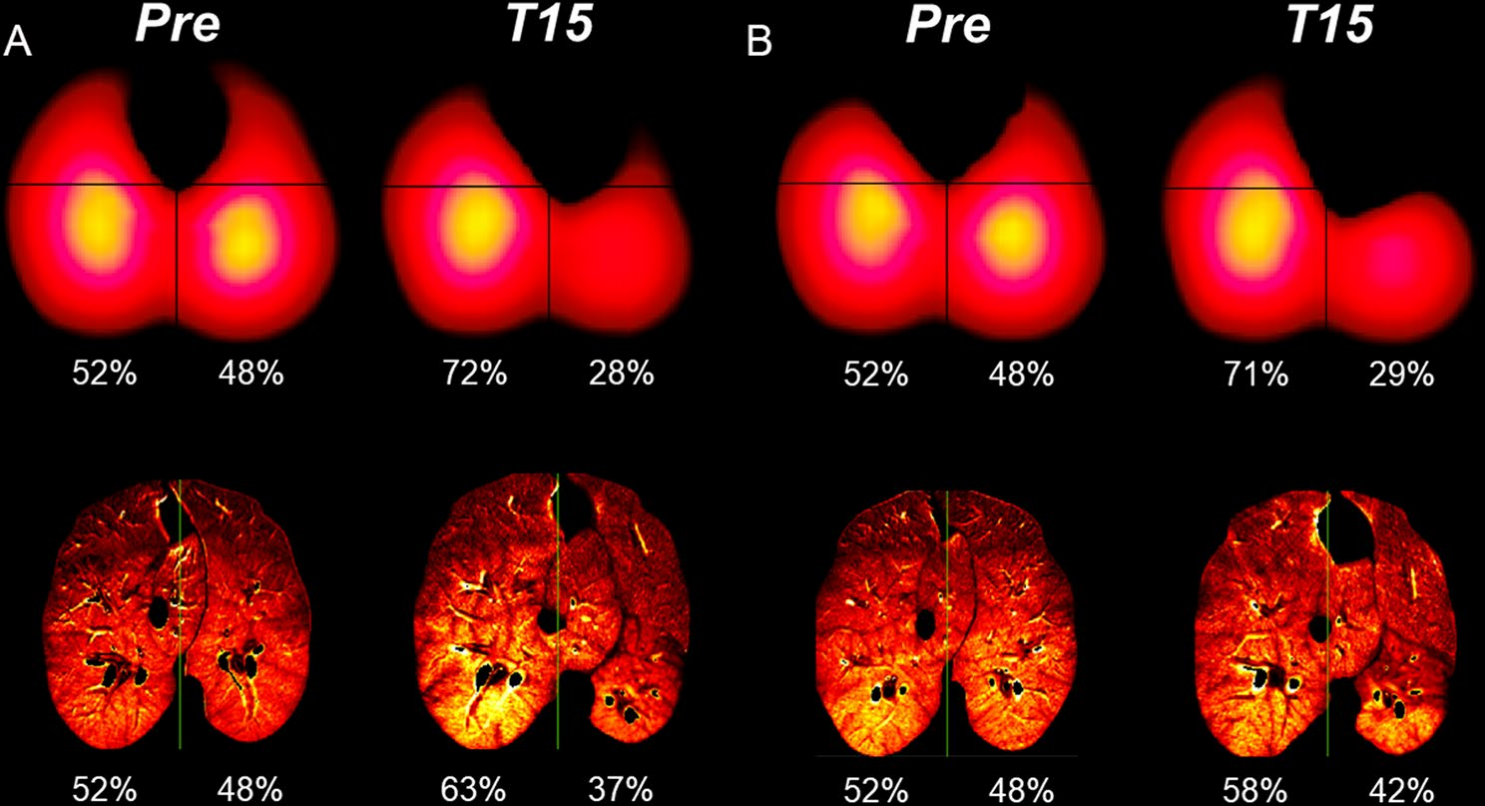
Perfusion distributions. Electrical impedance tomography (EIT) and dynamic contrast-enhanced computed tomography (CT) perfusion distributions with *Balloon* (**A**) and *Valves* (**B**) under fraction of inspired oxygen 1.0. ***Pre*** (under the *standard mechanical ventilation settings*) and ***T15*** (15-minute occlusion). The EIT and CT images were sub-segmented into two regions-of-interest (ROIs): right and left hemithoraces. Both imaging techniques showed a decreased perfusion in the left ROI at ***T15***

The dynamic contrast-enhanced CT perfusion and EIT perfusion distributions, acquired at steps ***Pre*** and ***T15*** in this animal, presented an apparent decrease of perfusion within the left ROI at ***T15*** (Fig. 4). There were no clear differences between the two F_I_O_2_.

EIT perfusion distributions, acquired at steps ***Pre*** and ***T45*** in five animals and ***Pre*** and ***T15*** in one animal (the one studied by CT): decreased significantly between ***Pre*** and ***T45***/***T15***, with no difference between the F_I_O_2_.

### Local pocket pressure

**Fig. 5:**
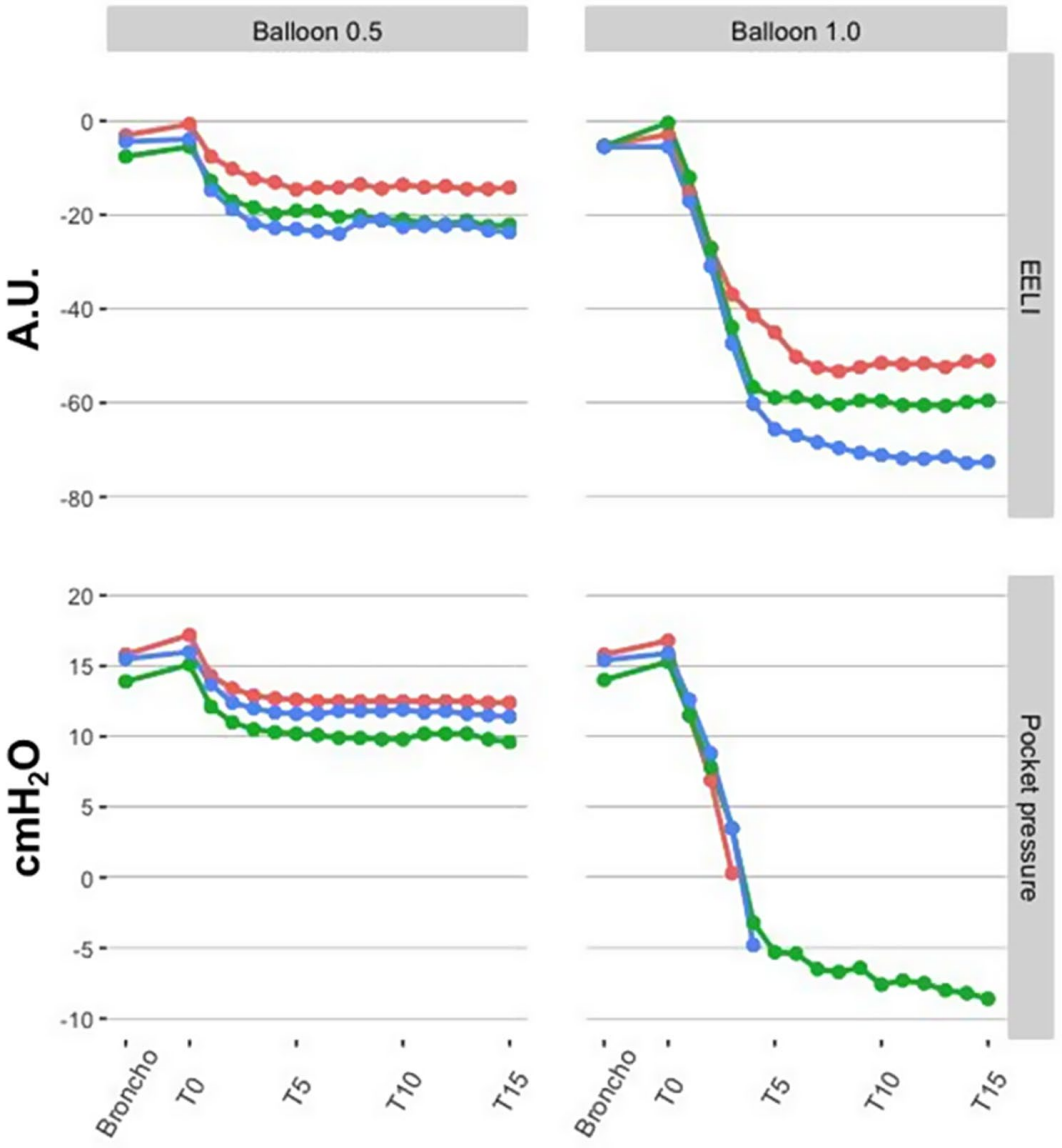
Regional end-expiratory lung impedance and local pocket pressure. During the *Balloon Occlusion Method*, a pressure transducer was connected to a hollow catheter to provide continuous recordings of the pressure changes in the air pocket distal to the occluded bronchi. The corresponding regional end-expiratory lung impedance (EELI) and the distal air pocket pressure changes are depicted. Data were acquired simultaneously in fractions of inspired oxygen (F_I_O_2_) 0.5 and 1.0. The local pocket pressure and its corresponding regional EELI paralleled each other. The negative local pocket pressure under F_I_O_2_ 1.0 was detected at 4 min and thereon in one animal with the longest stable measurement A.U: arbitrary units. Every line (with its corresponding color) is from an individual animal

The corresponding regional EELI and the pressure changes of the air pocket distal to the balloon occlusion are depicted in Fig. 5. Data were acquired simultaneously in both F_I_O_2_ regimens. The local pocket pressure and its corresponding regional EELI paralleled each other. Noteworthy, a negative local pocket pressure was obtained at 4 min and thereon under F_I_O_2_ 1.0 in one animal with the longest stable measurement.

### Patient data

Two patients were submitted to BLVR with EBV. The EIT imaging was obtained during the BLVR procedure. Additional details on the patient data are provided as Supplementary Information (See Supplementary Information S1, Additional File 1).

**Fig. 6:**
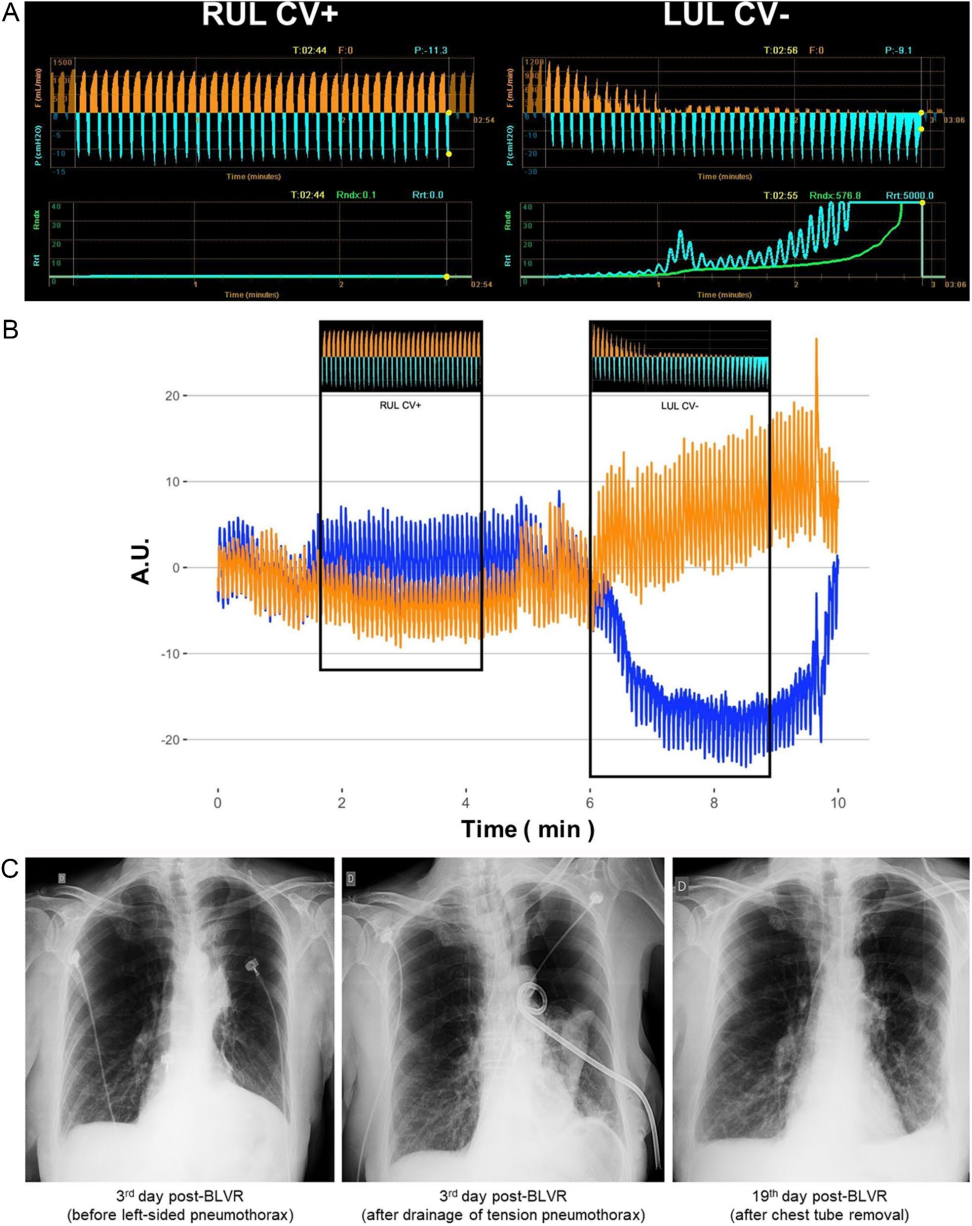
Observations from the first patient. A 62-year-old female with emphysema eligible for bronchoscopic lung volume reduction (BLVR) underwent a collateral ventilation (CV) assessment of the upper lobes with a Chartis™ catheter inserted into the working channel of the bronchoscope. The catheter was then advanced initially into the right upper lobe (RUL) bronchus (A; left) and after into the left upper lobe (LUL) bronchus (A; right) and inflated in each lobar bronchus until complete occlusion was achieved. Real-time electrical impedance tomography (EIT) imaging was continuously acquired to visualize changes in regional ventilation, and in regional end-expiratory lung impedance. Continuous tracings of the expiratory flow (F), inspiratory pressure (P), and resistance (R) of the corresponding occluded lobar bronchus were recorded by the Chartis™ console (**A**). The absence of CV (CV-) created a gradual decrease in the expiratory flow associated with a simultaneous gradual increase in the negative pressure and the airway resistance (A, right: LUL CV-). Such findings became apparent within the first minute of the balloon occlusion of the target lobe (A, right: LUL CV-). The anesthesiologist set the fraction of inspired oxygen to 0.8, and the EIT acquisition started (**B**) during the assessment of the RUL, followed by the LUL. The patient was then treated with four EBV-Zephyr™ valves deployed in the LUL’s segmental bronchi. (**B**): During the CV assessment of the RUL, with the Chartis™ measurements demonstrating a CV + pattern, there was an unremarkable variation of the regional EIT tracings (regional ventilation and regional end-expiratory lung impedance). Conversely, during the LUL Chartis™ assessment, the measurements demonstrated a CV- pattern corresponding to the synchronous real-time reduction in the blue regional impedance plethysmography in the left hemithorax region of interest. On the third day post-BLVR (**C**) the patient developed sudden respiratory distress due to a left-sided tension pneumothorax. The patient underwent an emergency bedside left chest tube drainage (**C**), leading to clinical stabilization but with a persistent air leak. The valve of the lingular bronchus was removed endoscopically on the 5th day post-BLVR, but the high-output air leak remained. On the 7th day post-BLVR, the patient underwent surgical repair of a ruptured bulla in the superior segment of the left lower lobe. The air leak resolved, allowing the chest tube removal and hospital discharge on the 19th day post-BLVR (**C**). CV+: presence of collateral ventilation; CV-: absence of collateral ventilation; RUL: right upper lobe; LUL: left upper lobe; blue regional impedance plethysmography: left hemithorax region-of-interest; orange regional impedance plethysmography: right hemithorax region-of-interest; A.U.: arbitrary units

*First Patient* (Figure. 6).

A 62-year-old female with emphysema and eligible to undergo BLVR treatment was submitted to a preprocedural bronchoscopy for CV assessment to define the target lobe. Under conscious sedation, topical anesthesia of the larynx, trachea, and bronchi and spontaneous breathing, a therapeutic flexible videobronchoscope advanced into the airway through a laryngeal mask. A Chartis™ catheter with a balloon tip was inserted into the bronchoscope working channel, advanced into the target left upper lobe bronchus, and inflated to complete bronchial occlusion. A continuous tracing of the pressure and expired flow of the occluded lobar bronchus was recorded by a Chartis™ console. The absence of CV created a gradual decrease in expiratory flow with an increase in the negative pressure and airway resistance. Such findings became apparent within the first minute of the balloon occlusion of the target lobe. The EIT acquisition started during the assessment of the right upper lobe, followed by the assessment of the left upper lobe. The patient received four EBV-Zephyr™ valves in the left upper lobe.

**Fig. 7:**
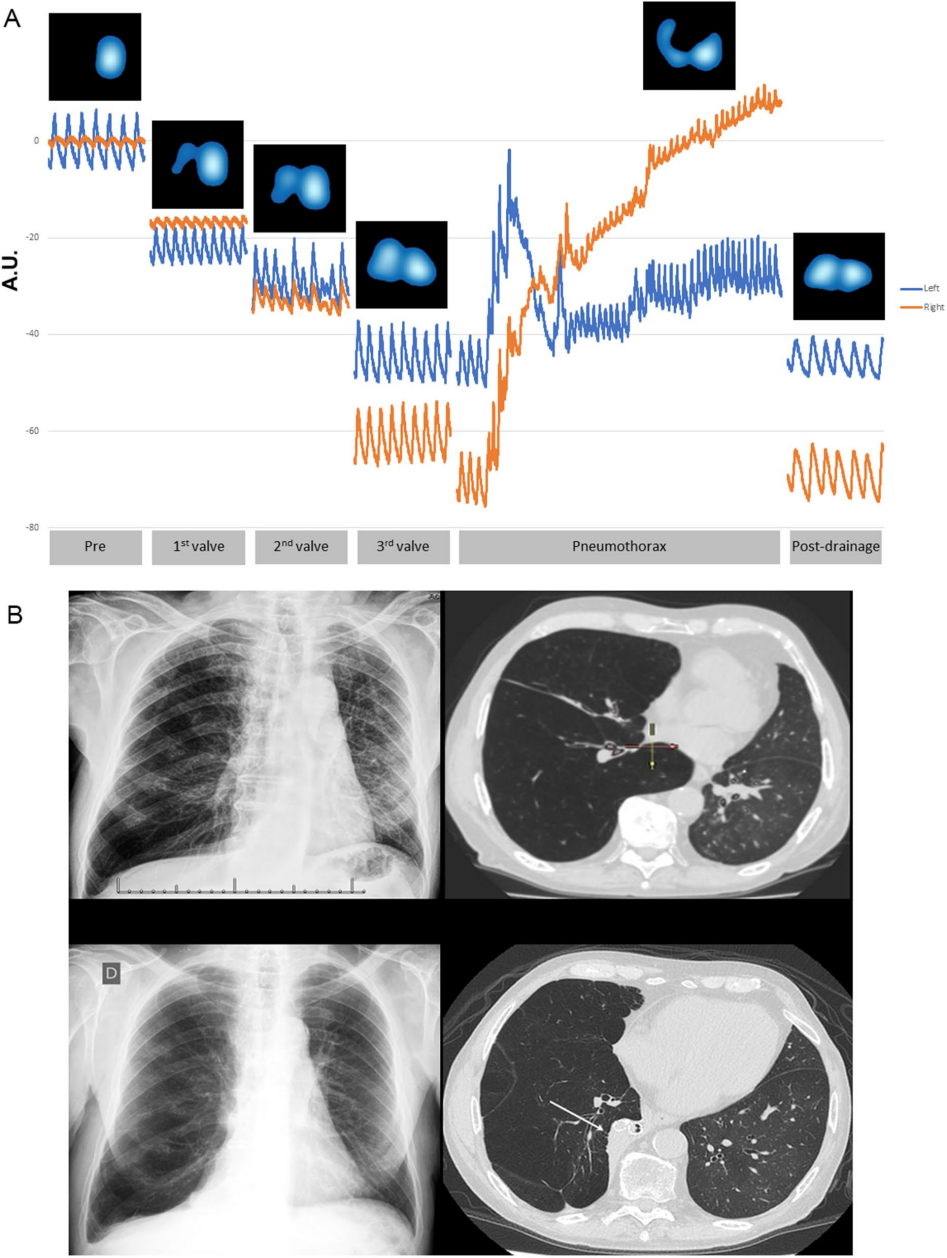
Observations from the second patient. A 71-year-old male with emphysema underwent a left single lung transplant seven years before. He developed progressive disabling dyspnea starting five years after transplantation, mainly because of progressive hyperinflation of the native lung. The forced expiratory volume in one second (FEV1) dropped from 1860 mL to 580 mL, and the post-transplant Modified Medical Research Council (mMRC) dyspnea scale shifted from 1 to 3 within the last year when he became full-time oxygen-dependent because of bronchiolitis obliterans and severe hyperinflation of the native lung. The observations from this patient focused on the periods before (briefly after sedation: Pre), during, and after bronchoscopic lung volume reduction (BLVR) with one-way endobronchial valves (EBV)-Zephyr^TM^ (See Supplementary Video S1, Additional File 5). Electrical impedance tomography (EIT) continuous real-time imaging showed the changes in regional ventilation, and changes in regional end-expiratory lung impedance (EELI). The EIT images were sub-segmented into two regions-of-interest (ROIs): right (orange) and left (blue) hemithorax. A Chartis^TM^ catheter was advanced into the right lower lobe bronchus, and a negative collateral ventilation (CV-) pattern was recorded by the Chartis^TM^ console. Three EBV Zephyr^TM^ valves were placed in the right lower lobe segmental bronchi. Upon completion of the BLVR procedure following the withdrawal of the laryngeal mask during recovery from the sedation, with the patient still in the operating room, he had a sudden coughing spell followed immediately by dyspnea, right-sided chest pain, and a steep decrease in peripheral capillary oxygen saturation (SpO2), from 98 to 80%. Immediately before the sudden coughing spell (See Supplementary Video S1, Additional File 5), the EIT tracings showed a quick rise in the regional EELI of the right hemithorax (orange EIT tracing), combined with a corresponding significant attenuation of the regional ventilation within the same right ROI (**A**), which are the typical changes of a pneumothorax in the EIT signals [23]. As the patient?s respiratory condition rapidly deteriorated, becoming critical, in addition to the accompanying real-time EIT tracings and images for pneumothorax altogether, allowed us to proceed with an emergency right-sided chest tube drainage. It was followed by improved dyspnea and pain, yielding stabilization of the ventilatory condition and SpO2 within a few minutes. (**A**) shows the EIT tracings and images of the events before (briefly after sedation: Pre), during each valve placement, and throughout the pneumothorax event, including post-drainage (See Supplementary Video S1, Additional File 5). The patient recovered in the operating room, and the air leak from the chest tube resolved gradually until its removal on the fifth day after the drainage procedure. Three months after the BLVR procedure, SpO2 increased from 89.6 to 93%, the FEV1 and the forced vital capacity (FVC) showed an increase of 130 mL and 250 mL, respectively. The dyspnea scale dropped from mMRC 3 to 2.(**B**): Chest radiograph and computed tomography scan of this patient showed in the upper panel (before BLVR) the hyperinflated right lung, primarily due to the right lower lobe displacing the mediastinum towards the left side. In the lower panel (three months after BLVR), the right lower lobe atelectasis with the endobronchial valves in place (arrow) and the mediastinal shifted towards the midline

*Second Patient* (Fig. 7; See Supplementary Video S1, Additional File 5).

Anesthetic and endoscopy procedures were like those described for the first patient. A Chartis™ catheter was advanced into the right lower lobe bronchus of a 71-year-old male with emphysema, and CV- was recorded by the Chartis™ console. Three EBV Zephyr™ valves were placed in the right lower lobe segmental bronchi. Upon completion of the BLVR procedure and withdrawal of the laryngeal mask during recovery from the sedation with the patient in the operating room, he had a sudden coughing spell followed immediately by dyspnea, right-sided chest pain, and a steep decrease in peripheral capillary oxygen saturation (SpO_2_), from 98 to 80%. Immediately before the sudden coughing spell, the EIT tracings showed a quick rise in the regional EELI of the right hemithorax, combined with a corresponding significant attenuation of the regional ventilation within the same right ROI, which are characteristic changes in the EIT signals suggestive of a pneumothorax [23]. As the patient’s respiratory condition rapidly deteriorated, becoming critical, in addition to the accompanying real-time EIT tracings and images for pneumothorax altogether, allowed us to proceed with an emergency right-sided chest tube drainage. It was followed by an improvement of the dyspnea and pain, yielding to stabilization of the ventilatory condition and SpO_2_ within a few minutes.

## Discussion

To our knowledge, this is the first study using EIT imaging to examine the real-time effects of high vs. low F_I_O_2_ during BLVR with EBV. We purposely selected a pig model that is CV- [31] because it simulates the ideal conditions for BLVR with one-way valves. In addition, the pig has a severe hypoxic pulmonary vasoconstriction (HPV) reflex that helps in its ventilation-perfusion (V̇/Q̇) balance [32]. Under such conditions, high F_I_O_2_ significantly amplifies the acute dynamics of lung volume reduction.

The foundations of BLVR explored the sustained lobar atelectasis to promote lung volume reduction without surgery [33]. In a landmark elegant study in a sheep model of emphysema produced by inhalation of papain, the authors demonstrated alveolar instability induced by F_I_O_2_ 1.0 for 15 min before occlusion and wash out of the surfactant within the target segment. There are similarities between that study and our present study regarding the underlying mechanisms and interventions. Ingenito and colleagues [33] found that the obliteration of dysfunctional regions of the hyperinflated lung could be achieved nonoperatively by a bronchoscopic production of sustained collapse by the following procedures: (a) filling target regions with oxygen, benefiting from its high absorption coefficient, which promotes reabsorption atelectasis; (b) using an airway occlusion method; in our case with either our inflatable balloon or EBV whereas in their case by the injection of a biocompatible fibrin-based glue acting as a sealant. In the last statement of their seminal paper, the authors encouraged a more highly refined approach to BLVR. Our investigation moves one step further in this direction.

Alveolar collapse has been associated with hypoventilation and airway closure during general anesthesia. The prevention of atelectasis has been attempted with the use of a titrated PEEP [34] or by lower F_I_O_2_, particularly before the induction of anesthesia [14–16, 35]. Nitrogen is a break for the gas absorption within the air pocket generated by airway occlusion. It increases the time to achieve atelectasis from as low as 4 to 6 min with F_I_O_2_ 1.0, to 6 to 8 h with F_I_O_2_ 0.21 [35, 36]. The driving force of gas absorption rate is a function of the pressure gradient between alveolar and mixed-venous gas concentrations, the absorption coefficient of each gas, and local blood flow [35–37]. Our data showed a larger EELI decrease with F_I_O_2_ 1.0. Notwithstanding, during balloon occlusion, approximately 90% of the lobar volume reduction occurred within the first 5 min in both F_I_O_2_ regimens (Fig. 1). It reflects the initial higher absorption rate of oxygen that decelerates proportionally as the concentration gradient decreases.

The CT images and data supported the EIT findings. It shows that the steepest fall in the regional CT-derived gas content achieved with F_I_O_2_ 1.0 is likely derived from the exhaustion of all gas content within the occluded segment. Conversely, under F_I_O_2_ 0.5 the reduction of the gas content does not necessarily result in atelectasis in the short term because nitrogen still supports the alveoli (Fig. 3). The regional CT-derived gas content and regional EELI findings data presented a notable matching (Fig. 2). Likewise, the local pocket pressure and EELI data showed an apparent matched behavior over time. The F_I_O_2_ 1.0 yielded a steeper decay in volumes and pressures when compared to F_I_O_2_ 0.5, reaching sub atmospheric pressures. These negative pressures likely indicate negative pleural/interstitial pressures created by interdependence forces generated by extreme volume reduction of the target lobe as compared to the surroundings (Fig. 5). This situation mimics the final result of a successful valve much more than the results at F_I_O_2_ 0.5.

Concerning such dynamics of volume reduction resulting from the gas absorption following lobar occlusion, the findings reinforce that F_I_O_2_ 1.0 yields a faster and more significant volume reduction than F_I_O_2_ 0.5, which relies on nitrogen to slow down the atelectasis induction once oxygen is absorbed. While EELI detected the dynamics of the occluded area, TIV expressed the behavior of the non-occluded lung regions. Our results showed a dissociation between the two. The EELI was both F_I_O_2_ and time-dependent, whereas the TIV changes were immediate and neither dependent on F_I_O_2_ nor its effects on EELI within the region. Of note, regional ventilation of the non-occluded areas increased immediately after balloon or valve occlusion.

The decrease in perfusion distribution found with EIT and CT perfusion measurements after 15 min of occlusion of the LLL, regardless of F_I_O_2_, corroborates the hypothesis of complete oxygen consumption followed by regional hypoxia within occluded areas. A strong HPV reflex was described previously in piglets to be triggered by local partial pressure of oxygen between 25 and 50 mmHg [38]. Perfusion distribution in emphysema varies widely, considering the increase of CV as an adaptive resource for V̇/Q̇ adjustments [39], in addition to the inverse relation between CV and HPV reflex [40]. In patients submitted to BLVR with EBV, V̇/Q̇ scintigraphy evaluations pre- and post-intervention showed a proportional reduction of perfusion in the treated lobe and a contralateral compensatory increase [41]. Thomsen and colleagues [42] showed a positive correlation between high non-target ipsilateral lobe perfusion and 6 min-walk distance test.

Pneumothorax after BLVR is a feared complication following lobar atelectasis. This patient population has a high relative risk and an incidence between 18 and 34% in clinical trials [43]. Approximately 86% occur within the first 72 h post-BLVR [44], and it is a potentially life-threatening situation [45]. A randomized clinical trial with EBV Zephyr valves (Liberate Trial) [10] reported a 26.6% incidence of pneumothorax. Post-BLVR atelectasis is a surrogate for better functional outcomes, and pneumothorax does not influence long-term survival [46]. The predictors of pneumothorax post-BLVR are based on static CT imaging data, including the presence of pleural-pulmonary adhesions, large volume of the ipsilateral untreated lobe over the volume of the hemithorax (whose effects on it are real-time detectable by EELI monitoring from EIT), emphysema type, fissure integrity, poor lung function, and exercise testing. Such factors yield a high probability of post-BLVR pneumothorax (84%) [12].

Egenod and Born [47, 48] proposed and performed a sequential placement of EBV-Zephyr valves in the target lobe by splitting the procedure into two stages one month apart. All but one endobronchial valve are placed in the first stage, and the last and more proximal valve is placed four weeks later. This strategy decreased the incidence of post-BLVR pneumothorax from 25 to 9%. Nevertheless, this BLVR with staged valve placement procedure has not yet reached a consensus due to the increased risk of bronchoscopy-related complications [49], and awaits a proper randomized controlled trial.

Lentz and colleagues hypothesized that a lower F_I_O_2_ during BLVR with EBV could slow absorption atelectasis by preventing nitrogen wash-out of the treated lung and reducing the incidence of pneumothorax [50]. The authors demonstrated that a low F_I_O_2_, compared with high F_I_O_2_ during BLVR with EBV was associated with a marked reduction in the incidence of post-procedure pneumothorax [50]. Furthermore, approximately 22% of the pneumothoraces in the high F_I_O_2_ group occurred during the first hour after the procedure thus supporting that atelectasis drives pneumothorax in high F_I_O_2_ patients. Our study may challenge this perspective for two reasons: first, the low F_I_O_2_ may decrease the chances of pneumothorax but, at the same time, they might decrease the efficiency and impact of each valve, as suggested in Fig. 1; and secondly, instead of promoting a slower reabsorption, we might propose a faster one, mimicking the full, long-term effect of a single valve, but monitoring the impact in real-time. This could result in fewer valves in place, but each of them with a maximized impact.

Based on the EIT’s ability to detect regional lung volume changes and ventilation shifts in real-time, we hypothesized that its use during the BLVR procedure could show the ventilation shifts that occur immediately after the procedure and potentially anticipate post-procedural pneumothorax, as shown in the two patient observations described.

### Study limitations

The CT data was obtained only in one animal as to illustrate and demonstrate lung volume reduction. The EIT-based regional analysis are both bidimensional (data are provided by electrodes placed in a single plane representing the axial view), and tridimensional (the finite elements mesh model assumes a 3-D propagation of current and voltage field). This results in an EIT cross-sectional slice with approximately 15 cm of thickness, although this may vary with the size and shape of the animal [51]. This property could potentially limit the detection of localized changes in lung volume occurring within the apical zones of the lung.

Despite the amount of data and the study’s consistent findings, data came from a few animals with healthy lungs. We were expecting more pronounced effects in the contralateral EELI (i.e. a contralateral increase), as observed in two animals, but not in all. The lack of consistent effects in the contralateral EELI, as opposed to ipsilateral non-target lung regions, can derive from normal lung compliance and less parenchymal heterogeneity, as opposed to an emphysematous lung. Of note, although the EIT data did not show a contralateral volume increase, the CT images show a considerable expansion of the accessory lobe, displaced towards the left hemithorax.

Lastly, the right and left hemithoraces (ROIs) segmentation used a division based on the mediastinum midline. The invasion of the left ROI following the volumetric increase of the accessory lobe may have decreased the left ROI sensitivity. Such limitation derives from the EIT spatial reconstruction algorithms and the challenging anatomical segmentation of individual lobes.

### Clinical implications

The development of lung volume reduction procedures identified several physiological mechanisms [33, 52]. In patients with severe emphysema eligible to BLVR with EBV valves, the best results are related to the absence of significant CV between the target and ipsilateral lobe(s), resulting in lobar atelectasis and, thereby, an effective lung volume reduction [4]. On the other hand, as illustrated by the current study, the onset of complete lobar atelectasis of the treated lobe triggers a fast and immediate intrathoracic negative pressure and non-target lobe hyperdistention that increases the chance of pneumothorax. We tested the impact of different F_I_O_2_ during this procedure as a proof-of-concept study in a well-controlled experimental setting. It showed that faster volume reduction can be achieved with higher F_I_O_2_, enhancing the final impact of EBV valves. Although the chances of pneumothorax could be potentially increased, the real-time monitoring with EIT would allow us to stop the procedure once a pre-specified volume reduction and ventilation redistribution was achieved. This procedure would also favor the maximal impact of each valve in place.

We acknowledge that the impact of other variables such as PEEP and MV mode, or the decision to maintain or not the patient’s respiratory drive during mechanical ventilation [4] must be considered. The study results are the basis for future clinical studies addressing EIT imaging to monitor and manage these complex, challenging, and variable patients in real-time.

EIT emerges as a non-invasive technology able to provide meaningful information that can contribute to the safety of BLVR as a peri-procedural monitoring tool, particularly for intraoperative decision-making and early detection of complications such as pneumothorax.

## Conclusions

EIT monitoring demonstrated, in real-time, a significant and faster volume reduction in the occluded lung region under F_I_O_2_ 1.0 if compared to F_I_O_2_ 0.5. Moreover, EIT imaging also could track the behavior of ipsilateral non-target lung regions, such as an increase of regional ventilation of the ipsilateral non-target region, as an indicator of an increased risk and early detection of a pneumothorax. By providing continuous information on regional lung volume changes, EIT is a promising tool for intraprocedural decision-making during bronchoscopic lung volume reduction, which can potentially impact the decision-making between the single or sequential treatment of the target lobe(s) and the subsequent clinical outcomes.

### Electronic supplementary material

Below is the link to the electronic supplementary material.

Supplementary Material 1

Supplementary Material 2

Supplementary Material 3

Supplementary Material 4

Supplementary Material 5

## Data Availability

The datasets used and/or analysed during the current study are available from the corresponding author on reasonable request.

## Abbreviations

ANOVA: Analysis of variance
BLVR: Bronchoscopic lung volume reduction
CT: Computed tomography
CV: Collateral ventilation
CV-: Negative collateral ventilation
EBV: One-way endobronchial valves
EELI: End-expiratory lung impedance
EIT: Electrical impedance tomography
F_I_O_2_: Fraction of inspired oxygen
HPV: Hypoxic pulmonary vasoconstriction
LLL: Left lower lobe
MV: Mechanical ventilation
PEEP: Positive end-expiratory pressure
ROI(s): Region(s)-of-interest
SpO_2_: Peripheral capillary oxygen saturation
TIV: Tidal impedance variation
V̇/Q̇: Ventilation-perfusion
