# Supplementary material for: First real-time imaging of bronchoscopic lung volume reduction by electrical impedance tomography
Source: Respir Res. 2024 Jul 4;25:264. doi: 10.1186/s12931-024-02877-0 (PMC11225379; doi:10.1186/s12931-024-02877-0)
Supplement: Supplementary file 1 — Supplementary Material 1 [file 12931_2024_2877_MOESM1_ESM.docx]

**Supplementary Information on the Methods and Results**

**Methods**

This study was approved by the ethics committee for experimental studies of the Faculdade de Medicina da Universidade de Sao Paulo, Sao Paulo, Brazil (CEUA 200-12), and by the ethics committee for clinical studies (CAAe 43250215.0.1001.5327 and CAPPesq 0689-11).

**Animal Experiments**

Six healthy female Landrace piglets (29.9 ± 2.2 kg) were pre-anesthetized with an intramuscular solution (midazolam 0.5 mg/kg; ketamine 5mg/kg; acepromazine 0.1 mg/kg). The animals were then placed in the supine position, and an ear venous access was obtained for anesthesia induction with 3 mg/kg propofol (Propovan, Cristalia, Brazil). The animals underwent orotracheal intubation, and mechanical ventilation (MV) started using *standard MV settings*: pressure-controlled mode; airway driving pressure adjusted to a tidal volume of 10 mL/kg; respiratory rate of 20 breaths per minute; inspiratory/expiratory ratio of 1:2 (See Supplementary Figure S1, Additional File 2). Anesthesia and muscle paralysis were maintained by intravenous continuous infusion of 1.7 mg/kg/hour ketamine (Quetamina, Vetnil, Brazil), 0.3 mg/kg/hour midazolam (Midazolam, Cristalia, Brazil), 0.02 mg/kg/hour fentanyl (Fentanest, Cristalia, Brazil), and 0.4 mg/kg/hour pancuronium (Pancuron, Cristalia, Brazil). A tracheostomy was then performed, and a central venous access and an arterial line (via femoral artery puncture) were placed. A PICCO catheter (Gettinge, Göteborg, Sweden) was inserted in the arterial line, and connected to a PICCO*plus*^®^ monitor (PC8100-Pulsion Medical Systems AG, Germany) for invasive blood pressure measurements and arterial blood sample collection. A pulmonary artery catheter was positioned for central venous, pulmonary arterial, and pulmonary capillary pressure monitoring (Dixtal^®^ Portal DX 2020 monitor, Dixtal, Brazil).

The same lung recruitment maneuver was performed before a positive end-expiratory pressure (PEEP) titration as described below and just before the beginning of each one of the four combinations of the two fraction of inspired oxygen (F_I_O_2_), 0.5 and 1.0, and the two occlusion methods, *Balloon* and *Valves*, (See Supplementary Figure S1, Additional File 2). Such lung recruitment maneuver was executed with the following MV settings: pressure-controlled mode, airway driving pressure of 15 cmH_2_O, respiratory rate of 20 bpm, F_I_O_2_ of 1.0, and inspiratory/expiratory ratio of 1.1. The initial PEEP level of the maneuver was 25 cmH_2_O (applied for 30 seconds), then increased to 30 cmH_2_O (also applied for 30 seconds), and finally increased to 35 cmH_2_O applied for 60 seconds.

The optimum PEEP of each animal was individually titrated as follows: after the lung recruitment maneuver, an electrical impedance tomography (EIT) based decremental PEEP titration [1] was performed. The MV settings at this time were volume-controlled mode, tidal volume of 5 mL/kg, respiratory rate of 30 bpm, F_I_O_2_ of 1.0, and inspiratory/expiratory ratio of 1:2. The decremental stepwise PEEP levels went from 21 to 7 cmH_2_O with two cmH_2_O for each step at fixed intervals of 40 seconds in each level. At the end of the EIT based decremental PEEP titration procedure, the optimum PEEP was defined as the lowest PEEP level where the lung collapse was 0%.

In a crossover study design, all animals were submitted to the two occlusion methods, *Balloon* and *Valves*, and to the two F_I_O_2_ regimens, 0.5 and 1.0 (See Supplementary Figure S1, Additional File 2). The occlusion method order was fixed with *Balloon* first. However, the F_I_O_2_ order was randomized. In each animal, the randomized F_I_O_2_ order for the *Balloon* *Occlusion Method* was repeated for the *Valves Occlusion Method*.

The lung recruitment maneuver reversed any lung collapse at the end of each of the four combinations of F_I_O_2_ and the occlusion methods just before the beginning of the next combination. The *standard MV settings* were resumed after each recruitment maneuver.

The animals stayed in the supine position throughout the study. The animals were euthanized at the end of the study with boluses of sedatives followed by a 10 mL 19.1% KCl bolus.

*Balloon* *Occlusion Method*

Bronchial occlusion of the left lower lobe (LLL) was achieved by the placement of an indwelling inflatable Chartis^TM^ balloon-catheter (Chartis^TM^, Pulmonx Inc. USA) under bronchoscopic view (Olympus BFB 160, Tokyo, Japan). To be able to study only the effects of the two F_I_O_2_, we did not perform any aspiration that is usually made when using the Chartis^TM^ console. The balloon was held in place, occlusion was maintained for 15 minutes, and the balloon was deflated.

The following time points were analyzed: ***Pre*** (under the *standard MV settings*); ***Broncho*** (1 minute after positioning the bronchoscope in the LLL bronchus; this step was included to ensure that the bronchoscope did not cause any confounding effect); ***T0*** (when complete LLL bronchial occlusion with the balloon was achieved); and each minute from ***T1*** to ***T15*** (1 to 15 minutes of occlusion). This method required the bronchoscope to remain within the airway lumen during all the time points acquisitions because the balloon catheter is introduced through the working channel of the bronchoscope. Furthermore, it allowed the investigator to check visually that the occlusion was complete.

*Valves Occlusion Method*

The bronchoscopic placement of one-way endobronchial valves EBV Zephyr^TM^ 4.0 mm or 5.5 mm (Pulmonx Inc. USA) was carried out under bronchoscopic view and endobronchial valves were deployed in the segmental bronchi of the LLL. Upon completion of the valve placement and bronchoscope withdrawal, the animals were observed for 45 minutes, and the valves were removed with endoscopic forceps (Rat Tooth Grasping Forceps FG-26C-1, Olympus America USA).

The following time points were analyzed: ***Pre*** (under the *standard MV settings*); ***Broncho*** (1 minute after positioning the bronchoscope in the LLL bronchus); ***T0*** (when complete LLL bronchial occlusion with the valves was achieved); and each minute from ***T1*** to ***T15*** (1 to 15 minutes of occlusion); ***T30*** and ***T45*** (30 and 45 minutes of occlusion). This method removed the bronchoscope immediately after all valves were deployed and secured.

*EIT Imaging*

EIT continuously measured thoracic impedance variations at a rate of 50 Hz (Enlight 2100, Timpel Medical, Sao Paulo, Brazil). Extra-small electrode bands (60 mm-thick) were placed inferior to the piglet’s glenohumeral joint according to the manufacturer’s recommendation. A pneumotachograph was placed proximally to the endotracheal tube and connected to the EIT monitor. The data were then reconstructed into an image consisting of a 32 by 32-pixel spatial matrix [2].

The following functional lung images were generated by EIT:

*Regional Lung Volumes and Regional Ventilation*: They were derived from relative impedance changes, which reliably track local, pixel-by-pixel changes in the content of air within the lung [2-4]. Measurements consisted of the regional tidal impedance variation (TIV), which corresponds to the regional impedance amplitude during a tidal breath, and the regional end-expiratory lung impedance (EELI), which are the changes in the impedance plethysmography baseline, which is a surrogate for regional end-expiratory lung volume (EELV) [5, 6]. For such analyzes, continuous EIT data acquisition was carried out during both occlusion methods (*Balloon* and *Valves*) and both F_I_O_2_ (1.0 and 0.5). EIT data were relative to ***Pre*** values for each combination of F_I_O_2_ and occlusion method.

*Perfusion Distributions*: They were acquired in six piglets as previously described [7]. A bolus of 10% sodium chloride solution (10 mL) was injected over one second into a central venous catheter during 20 seconds of apnea in continuous positive airway pressure (CPAP) mode, using the PEEP level measured in the ventilator before the apnea. The impedance versus time waveform of each pixel was acquired and exported to a custom LabVIEW software (National Instruments, Austin, TX). Each pixel’s impedance versus time curve was analyzed for a biphasic model as previously described [7]. If two components were present, the first appearing component indicated pre-lung signal from the right heart phase or vascular tissue. The resulting lung curve was fit to a single gamma function, which was then reconstructed into a 32 by 32-pixel matrix overlapping the ventilation matrix. Using the first-pass kinetic method [7], the maximum slope of each pixel’s gamma function was calculated, yielding a relative regional perfusion map.

For all EIT data analysis, the EIT images were sub-segmented into two regions-of-interest (ROIs): *Right* and *Left* hemithorax.

*Computed Tomography Scans*

To compare with the EIT findings as well as to help with their interpretation and of the mechanisms involved, helical computed tomography (CT) scans (Discovery CT750 HD, GE Healthcare, USA) and EIT imaging were simultaneously acquired in one of the piglets.

For both occlusion methods and F_I_O_2_, the time points ***Pre***, each minute from ***T0*** to ***T5***, and ***T15*** were analyzed as regards to the CT-derived gas content.

In addition, dynamic-contrast enhanced CT perfusion distributions, as described in detail elsewhere [8, 9], were also acquired for both occlusion methods and F_I_O_2_ in this same one animal, at the time points ***Pre*** and ***T15***, having EIT perfusion distributions simultaneously acquired.

*Local Pocket Pressure*

During the *Balloon* *Occlusion Method,* a pressure transducer was connected to a hollow catheter to provide continuous recordings of the pressure changes in the air pocket distal to the occluded bronchi. This methodology has been described in detail previously [10, 11].

**Patients Observations**

The observations in the first patient focused on the collateral ventilation (CV) evaluation to define the target lobe where CV was absent. The observations of the second patient focused on the periods before, during, and after the EBV Zephyr^TM^ valve placement. In addition to the patients' clinical monitoring, EIT imaging was real-time and continuously acquired to visualize both the regional ventilation and the changes in regional EELI.

The EIT data were recorded at a rate of 50 Hz by the same equipment used for the experimental acquisitions (Enlight 2100, Timpel Medical, Sao Paulo, Brazil). A 32-electrode belt placed on the perimeter defining a cross-sectional plane of the thorax at the fifth to the sixth intercostal space level was attached. Similarly to the experimental data, measurements consisted of the regional TIV and regional EELI, and the images were sub-segmented into the same ROIs as the piglets: *Right* and *Left* hemithorax.

**Statistical Analysis**

The assumptions of a normal distribution in each group and the homogeneity of the variances between groups were evaluated with the Shapiro-Wilk and Levene tests. A two-way **analysis of variance** (ANOVA) was used for a two-factor analysis (group and time), and Bonferroni adjustment for multiple tests was applied for *post-hoc* comparisons. The paired-samples *t*-test was used to determine whether the mean difference between paired observations is statistically significantly different from zero. Statistical significance was considered for *p* values less than 0.05. Values presented are mean and SEM unless otherwise stated.

**Results**

**Experimental Data**

*Perfusions Distributions by EIT*

About only the EIT perfusion distributions, acquired at steps ***Pre*** and ***T45*** in five animals, and at steps ***Pre*** and ***T15*** in one animal (the one studied with CT scans), we have found the following results:

- *Balloon Occlusion Method*: decrease in the perfusion of the left ROI from 48.1 ± 5.2 to 35.6 ± 4.2% under F_I_O_2_ 1.0 (*p* = 0.005), and from 49.1 ± 3.7 to 39.9 ± 3.4% under F_I_O_2_ 0.5 (*p* = 0.001).

- *Valves Occlusion Method*: decrease in the perfusion of the left ROI from 48.2 ± 2.7 to 34.5 ± 6.5% under F_I_O_2_ 1.0 (*p* = 0.001), and from 49.4 ± 2.4 to 32.6 ± 8.6% under F_I_O_2_ 0.5 (*p* = 0.001).

Pooling together the second step acquisitions (***T45*** in five animals and ***T15*** in one animal) of the two occlusion methods, and comparing only the two F_I_O_2_, there was no significant difference in the EIT perfusion distributions: 35.1 ± 5.3 under F_I_O_2_ 1.0 to 36.3 ± 7.3% under F_I_O_2_ 0.5 (*p* = 0.352).

Pooling together the two occlusion methods, and comparing ***Pre*** vs. the second step acquisitions, under both F_I_O_2_, we found the following: 48.2 ± 3.9 to 35.1 ± 5.3% under F_I_O_2_ 1.0 (*p* < 0.0005); 49.2 ± 3.0 to 36.3 ± 7.3% under F_I_O_2_ 0.5 (*p* < 0.0005).

**Patient Data**

Two patients were submitted to bronchoscopic lung volume reduction (BLVR) with one-way endobronchial valves (EBV). The EIT imaging was obtained during the BLVR procedure.

*Observations from the First Patient*

A 62-year-old female with emphysema and eligible to undergo BLVR treatment was submitted to a preprocedural bronchoscopy for CV assessment to define the target lobe. Under conscious sedation, topical anesthesia of the larynx, trachea, and bronchi and spontaneous breathing, a therapeutic flexible videobronchoscope (Olympus BF1T 180, Olympus, Japan) advanced into the airway through a laryngeal mask. A Chartis^TM^ catheter with a balloon tip was inserted into the bronchoscope working channel, advanced into the target left upper lobe bronchus, and inflated to complete bronchial occlusion. A continuous tracing of the pressure and expired flow of the occluded lobar bronchus was recorded by a Chartis^TM^ console. The absence of CV created a gradual decrease in expiratory flow with an increase in the negative pressure and airway resistance. Such findings became apparent within the first minute of the balloon occlusion of the target lobe (Figure 6A). The anesthesiologist set F_I_O_2_ 0.8, and the EIT acquisition started (Figure 6B) during the assessment of the right upper lobe, followed by the assessment of the left upper lobe. The patient received four EBV-Zephyr^TM^ valves in the left upper lobe. On the third day post-BLVR the patient developed sudden respiratory distress secondary to a left-sided tension pneumothorax. The patient underwent an emergency bedside left chest tube drainage, leading to clinical stabilization but with a persistent air leak. The valve of the lingular bronchus was removed endoscopically on the 5^th^ day post-BLVR, but the high-output air leak remained. On the 7^th^ day post-BLVR, the patient underwent surgical repair of a ruptured bulla in the superior segment of the left lower lobe. The air leak resolved, allowing chest tube removal and hospital discharge on the 19^th^ day post-BLVR (Figure 6C).

*Observations from the Second Patient*

A 71-year-old male with emphysema underwent a left single lung transplant seven years before. He developed progressive disabling dyspnea starting in the fifth-year post-transplant due to progressive hyperinflation of the native lung. The forced expiratory volume in one second (FEV1) dropped from 1860 mL to 580 mL, and his post-transplant Modified Medical Research Council (mMRC) dyspnea scale shifted from 1 to 3 within the last year. He became full-time oxygen dependent because of bronchiolitis obliterans and severe hyperinflation of the native lung. Anesthetic and endoscopy procedures were like those described for the first patient. A Chartis^TM^ catheter was advanced into the right lower lobe bronchus, and negative collateral ventilation (CV-) was recorded by the Chartis^TM^ console. Three EBV Zephyr^TM^ valves were placed in the right lower lobe segmental bronchi. Upon completion of the BLVR procedure and withdrawal of the laryngeal mask during recovery from the sedation with the patient in the operating room, he had a sudden coughing spell followed immediately by dyspnea, right-sided chest pain, and a steep decrease in peripheral capillary oxygen saturation (SpO_2_), from 98 to 80%. Immediately before the sudden coughing spell (See Supplementary Video 1, Additional File 5), the EIT tracings showed a quick rise in the regional EELI of the right hemithorax (orange EIT tracing), combined with a corresponding significant attenuation of the regional ventilation within the same right ROI (Figure 7A), which are characteristic changes in the EIT signals suggestive of a pneumothorax [12], (See Supplementary Video 1, Additional File 5). As the patient’s respiratory condition rapidly deteriorated, becoming critical, in addition to the accompanying real-time EIT tracings and images for pneumothorax altogether, allowed us to proceed with an emergency right-sided chest tube drainage with a #14 pigtail chest catheter (Wayne Pneumothorax Catheter Set, Cook Medical, USA). It was followed by an improvement of the dyspnea and pain, yielding to stabilization of the ventilatory condition and SpO_2_ within a few minutes. The EIT tracings and images of the events before (briefly after sedation), during each valve placement, and throughout the pneumothorax event, including its drainage, are shown in Figure 7A and Supplementary Video 1, Additional File 5.

The patient recovered, and the air leak through the chest tube resolved gradually, allowing its removal on the fifth day after the drainage procedure. Three months after the BLVR procedure, SpO_2_ increased from 89.6 to 93%, the FEV1 and the forced vital capacity (FVC) showed an increase of 130 mL and 250 mL, respectively. The dyspnea scale changed from mMRC 3 to 2. The chest radiograph and CT scan three months after the valve occlusion of the right lower lobe showed complete atelectasis of the lobe, and the mediastinum shifted towards the midline (Figure 7B).

**References**

1. Costa EL, Borges JB, Melo A, Suarez-Sipmann F, Toufen C, Jr., Bohm SH, Amato MB: **Bedside estimation of recruitable alveolar collapse and hyperdistension by electrical impedance tomography.** *Intensive Care Med* 2009, **35:**1132-1137.

2. Victorino JA, Borges JB, Okamoto VN, Matos GF, Tucci MR, Caramez MP, Tanaka H, Sipmann FS, Santos DC, Barbas CS, et al: **Imbalances in regional lung ventilation: a validation study on electrical impedance tomography.** *Am J Respir Crit Care Med* 2004, **169:**791-800.

3. Frerichs I, Hahn G, Golisch W, Kurpitz M, Burchardi H, Hellige G: **Monitoring perioperative changes in distribution of pulmonary ventilation by functional electrical impedance tomography.** *Acta Anaesthesiol Scand* 1998, **42:**721-726.

4. Frerichs I: **Electrical impedance tomography (EIT) in applications related to lung and ventilation: a review of experimental and clinical activities.** *Physiol Meas* 2000, **21:**R1-21.

5. Hinz J, Hahn G, Neumann P, Sydow M, Mohrenweiser P, Hellige G, Burchardi H: **End-expiratory lung impedance change enables bedside monitoring of end-expiratory lung volume change.** *Intensive Care Med* 2003, **29:**37-43.

6. Frerichs I, Amato MB, van Kaam AH, Tingay DG, Zhao Z, Grychtol B, Bodenstein M, Gagnon H, Bohm SH, Teschner E, et al: **Chest electrical impedance tomography examination, data analysis, terminology, clinical use and recommendations: consensus statement of the TRanslational EIT developmeNt stuDy group.** *Thorax* 2017, **72:**83-93.

7. Borges JB, Suarez-Sipmann F, Bohm SH, Tusman G, Melo A, Maripuu E, Sandstrom M, Park M, Costa EL, Hedenstierna G, Amato M: **Regional lung perfusion estimated by electrical impedance tomography in a piglet model of lung collapse.** *J Appl Physiol (1985)* 2012, **112:**225-236.

8. Fieselmann A, Kowarschik M, Ganguly A, Hornegger J, Fahrig R: **Deconvolution-Based CT and MR Brain Perfusion Measurement: Theoretical Model Revisited and Practical Implementation Details.** *Int J Biomed Imaging* 2011, **2011:**467563.

9. Xin Y, Kim T, Winkler T, Brix G, Gaulton T, Gerard SE, Herrmann J, Martin KT, Victor M, Reutlinger K, et al: **Improving pulmonary perfusion assessment by dynamic contrast-enhanced computed tomography in an experimental lung injury model.** *J Appl Physiol (1985)* 2023, **134:**1496-1507.

10. Yoshida T, Torsani V, Gomes S, De Santis RR, Beraldo MA, Costa EL, Tucci MR, Zin WA, Kavanagh BP, Amato MB: **Spontaneous effort causes occult pendelluft during mechanical ventilation.** *Am J Respir Crit Care Med* 2013, **188:**1420-1427.

11. Morais CCA, Koyama Y, Yoshida T, Plens GM, Gomes S, Lima CAS, Ramos OPS, Pereira SM, Kawaguchi N, Yamamoto H, et al: **High Positive End-Expiratory Pressure Renders Spontaneous Effort Noninjurious.** *Am J Respir Crit Care Med* 2018, **197:**1285-1296.

12. Costa EL, Chaves CN, Gomes S, Beraldo MA, Volpe MS, Tucci MR, Schettino IA, Bohm SH, Carvalho CR, Tanaka H, et al: **Real-time detection of pneumothorax using electrical impedance tomography.** *Crit Care Med* 2008, **36:**1230-1238.
