# Supplementary figures and images for: First real-time imaging of bronchoscopic lung volume reduction by electrical impedance tomography
Source: Respir Res. 2024 Jul 4;25:264. doi: 10.1186/s12931-024-02877-0 (PMC11225379; doi:10.1186/s12931-024-02877-0)

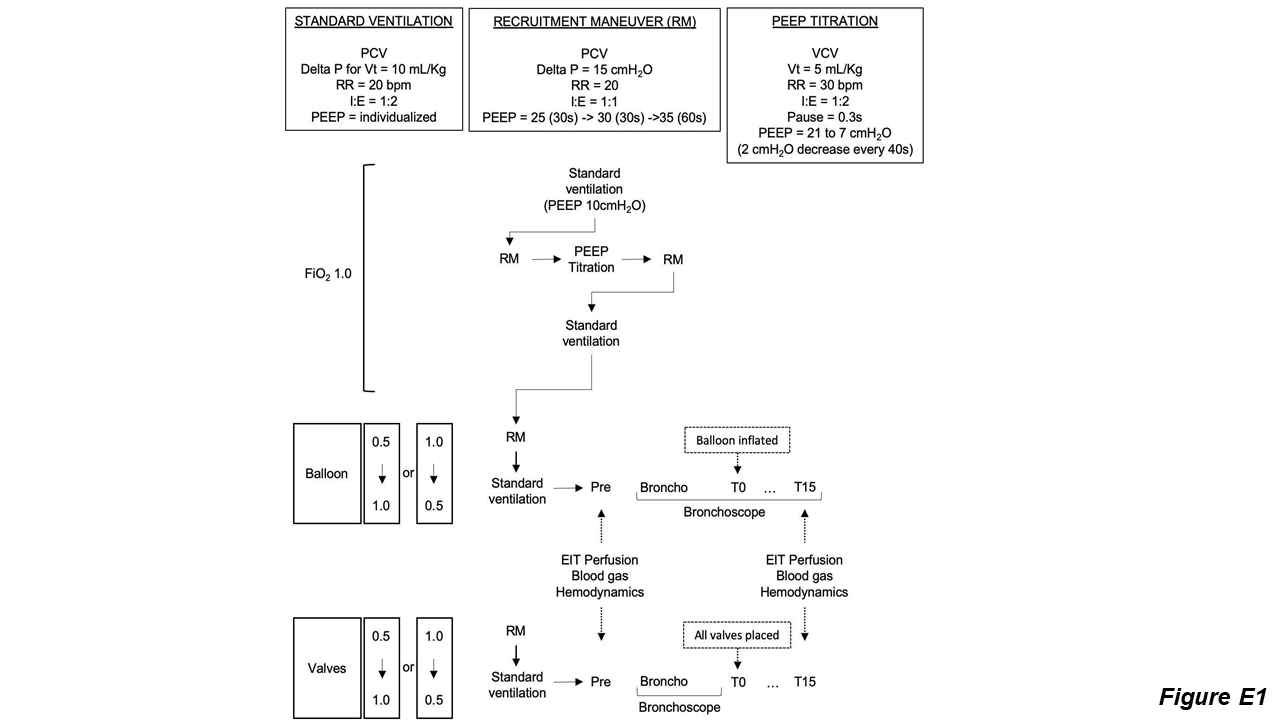

Supplement: Supplementary file 2 — Supplementary Material 2 [file 12931_2024_2877_MOESM2_ESM.tif]
