# Supplementary material for: First real-time imaging of bronchoscopic lung volume reduction by electrical impedance tomography
Source: Respir Res. 2024 Jul 4;25:264. doi: 10.1186/s12931-024-02877-0 (PMC11225379; doi:10.1186/s12931-024-02877-0)
Supplement: Supplementary file 3 — Supplementary Material 3 [file 12931_2024_2877_MOESM3_ESM.docx]

**Table S1: Mechanical Ventilation Parameters, Physiological Measurements and Respiratory System Mechanics – *Balloon Occlusion Method***

* *P* < 0.05 compared with the step ***Pre*** of the same F_I_O_2_.

F_I_O_2_: fraction of inspired oxygen
