# Supplementary material for: First real-time imaging of bronchoscopic lung volume reduction by electrical impedance tomography
Source: Respir Res. 2024 Jul 4;25:264. doi: 10.1186/s12931-024-02877-0 (PMC11225379; doi:10.1186/s12931-024-02877-0)
Supplement: Supplementary file 4 — Supplementary Material 4 [file 12931_2024_2877_MOESM4_ESM.docx]

**Table S2: Mechanical Ventilation Parameters, Physiological Measurements and Respiratory System Mechanics – *Valves Occlusion Method***

* *P* < 0.05, one-way repeated measures analysis of variance, for F_I_O_2_ 0.5.

**†** *P* < 0.05, one-way repeated measures analysis of variance, for F_I_O_2_ 1.0.

F_I_O_2_: fraction of inspired oxygen
